# A Surgical Conundrum in Feeding Jejunostomy–Jejunojejunal Intussusception: A Case Series

**DOI:** 10.7759/cureus.2233

**Published:** 2018-02-26

**Authors:** H Sakthivel, Ashok Kumar Sahoo, Anandhi Amaranathan, Nagarajan Raj Kumar, Nanda K Maroju

**Affiliations:** 1 MBBS, Ms Senior Resident, Jawaharlal Institute of Postgraduate Medical Education and Research (JIPMER), Puducherry, India.; 2 Surgery, Jawaharlal Institute of Postgraduate Medical Education and Research (JIPMER), Puducherry, India.

**Keywords:** intussusception, intestinal obstruction, feeding tubes

## Abstract

Intussusception is a common cause of intestinal obstruction in the pediatric population. Usually, it is primary and benign and can be managed by nonoperative interventions in 80% of the cases. Adult intussusception accounts for only 5% of all cases of intussusception and 1%–5% of all cases of intestinal obstruction. Unlike in the pediatric population, intussusception in adults is usually caused by a pathologic lead point. The initial investigation to diagnose it is an ultrasound abdomen followed by contrast-enhanced computed tomography (CECT) of the abdomen. The placement of an intestinal tube for feeding purposes has been rarely reported as a cause of intussusception. Here, we present a case series of four patients who had jejunojejunal intussusception following the placement of feeding tubes into the jejunum. Three patients were operatively managed and one was managed conservatively.

## Introduction

Intussusception of the bowel is defined as the telescoping of a segment of the bowel into the adjacent segment. It is one of the most common causes of bowel obstruction in infants and toddlers [1]. In intussusception, a portion of the proximal bowel (i.e., intussusceptum) invaginates into a more distal segment of the bowel (i.e., intussuscipiens). Most commonly, intussusception occurs in the pediatric population and is idiopathic. It is speculated that the hypertrophy of the Peyer’s patches may result in intussusception. On the other hand, adult intussusception is a rare entity and accounts for 5% of all cases of intussusception and 1%-5% cases of intestinal obstruction [2]. In about 90% of the cases in adults, there is a definitive organic lesion present, which causes intussusception. Depending upon the site of the lead point, they can be classified into entero-enteric, ileocolic, ileocecal, and colo-colic [3]. Among those cases, the postoperative intussusception is a special entity that can be idiopathic or may be associated with mucosal, intramural, or extrinsic causes. The various causes described in literature leading to the problem are polyps, lipoma, carcinoids, Meckels’ diverticulum, melanoma metastases, lymphomas, suture lines, adhesions, submucosal bowel edemas, intestinal dysmotility, long intestinal feeding tubes, and chronic dilatation of the bowel [4]. Adult intussusception is usually associated with a lead point and needs surgical intervention. The placement of a long intestinal tube for feeding purposes has been very rarely reported as a cause for intussusception. Here, we present a case series of four adult intussusceptions caused by the surgical placement of feeding tubes and discuss their management strategies.

## Case presentation

Case 1

**Figure 1:**
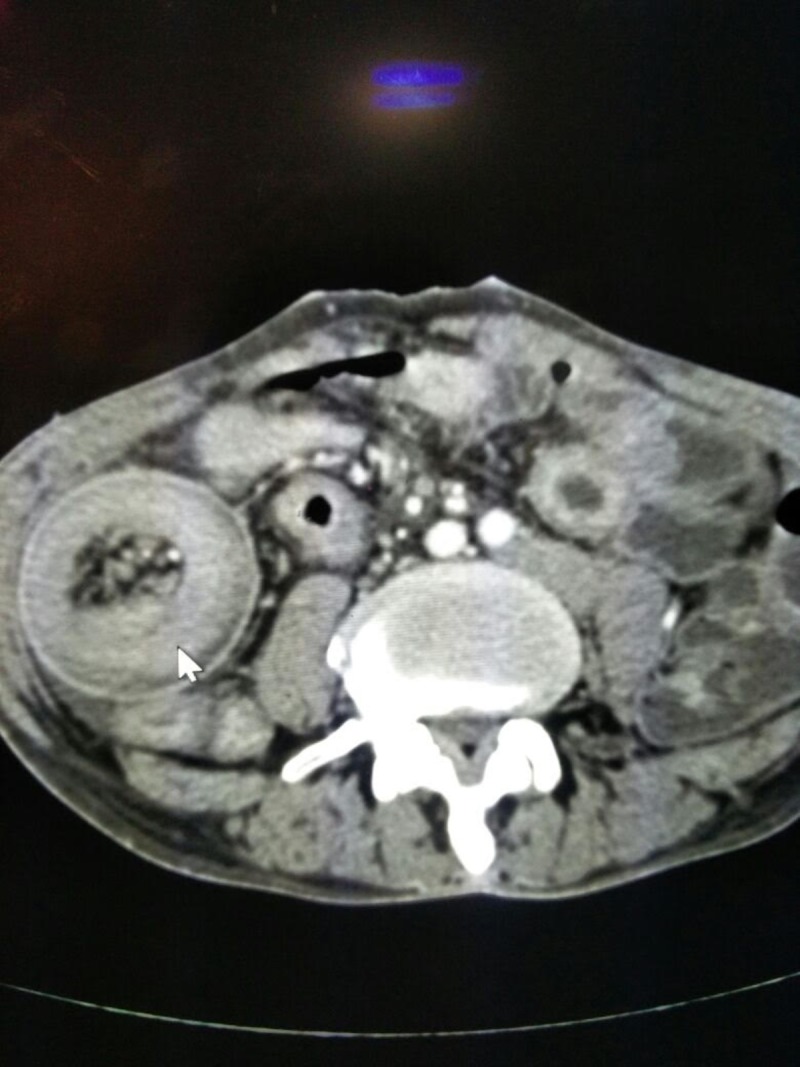
CECT showing the target sign suggestive of intussusception around the feeding tube

A 48-year-old patient presented with absolute dysphagia. An upper gastrointestinal endoscopy (UGIE) revealed an ulceroproliferative growth in the cervical esophagus, 18 cm from the incisors. The biopsy from the growth showed moderately differentiated squamous cell carcinoma. The contrast-enhanced computed tomography (CECT) thorax and abdomen showed the cardiac apex, descending arch of aorta, spleen, and stomach on the right side and liver on the left side suggestive of situs inversus totalis. There was a circumferential polypoidal growth involving the cervical esophagus from C5 to T3 with the loss of fat planes with the trachea. The patient was planned for chemoradiotherapy and in view of absolute dysphagia, laparotomy, and feeding jejunostomy (FJ) was done by the Witzels technique using 14 French (Fr) nasogastric tube. Postoperatively, after starting jejunostomy feeds, the patient developed intermittent colicky type abdominal pain and abdominal distension. The contrast study through the FJ tube showed suspected intussusception, which was confirmed with a CECT of the abdomen (Figure 1).

**Figure 2:**
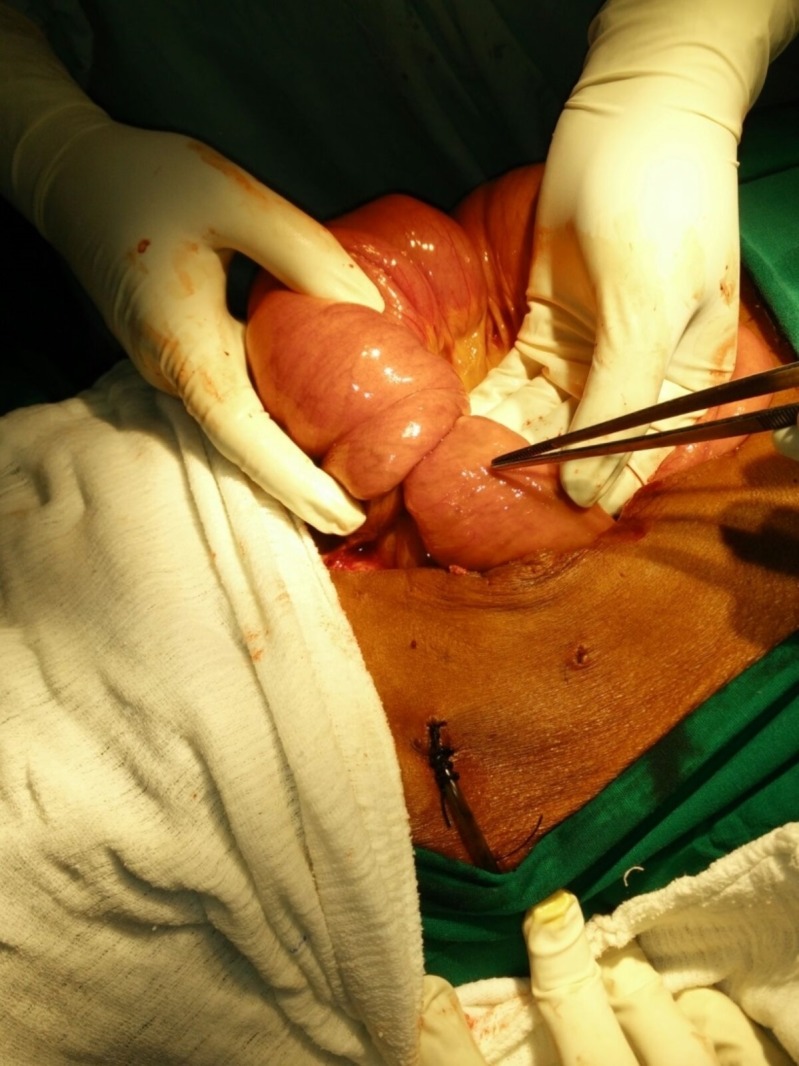
Jejunojejunal intussusception around the feeding jejunostomy on exploration

**Figure 3:**
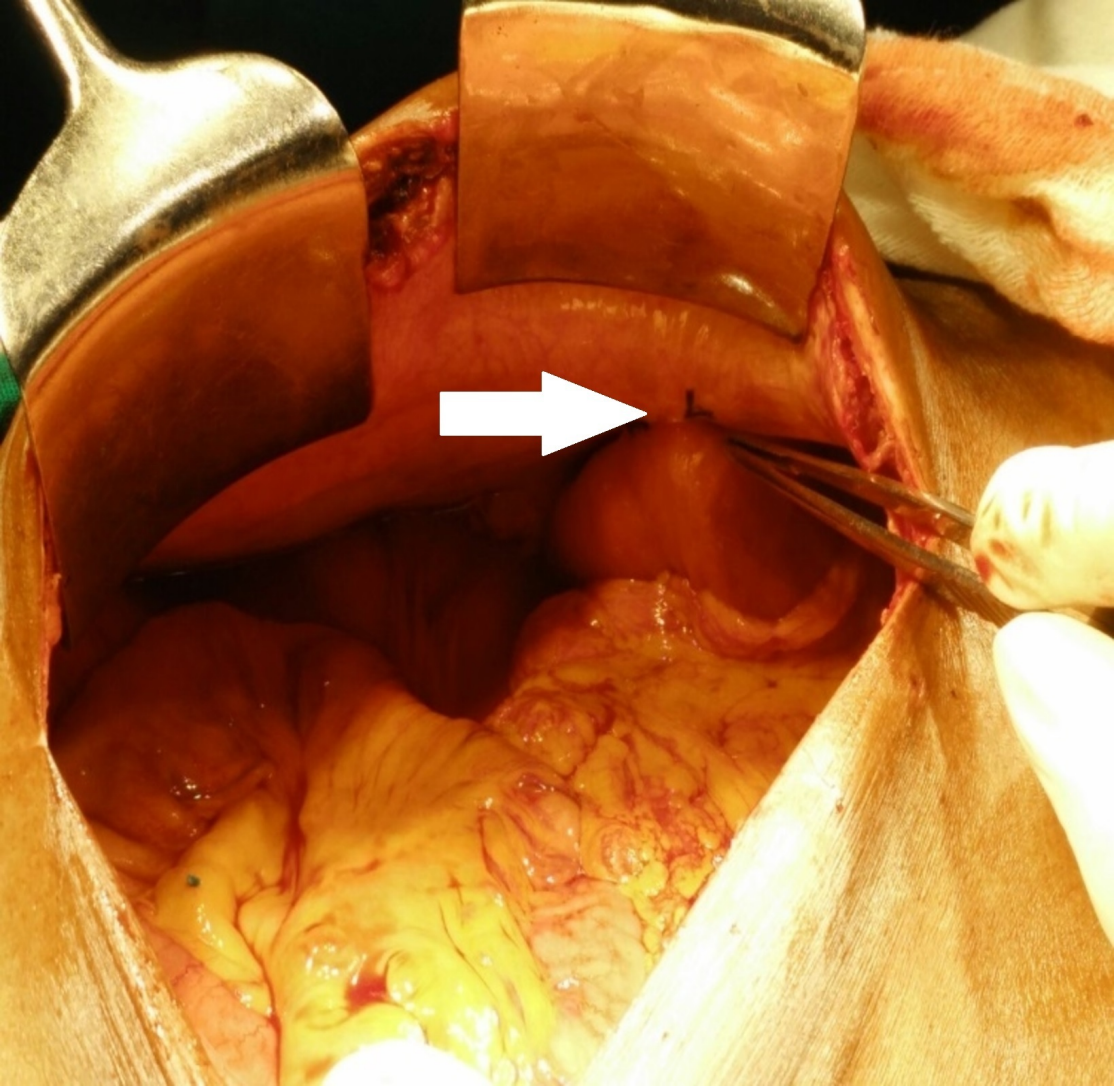
Jejunal loop fixed to the parietal wall found to be intact without any kink (white arrow)

On re-exploration, there was a jejunojejunal intussusception and the tip of the FJ tube was found to be the lead point (Figures 2-3).

The jejunojejunal intussusception was reduced. Postoperatively, the patient had an uneventful recovery and tolerated jejunostomy feeds.

Case 2

**Figure 4:**
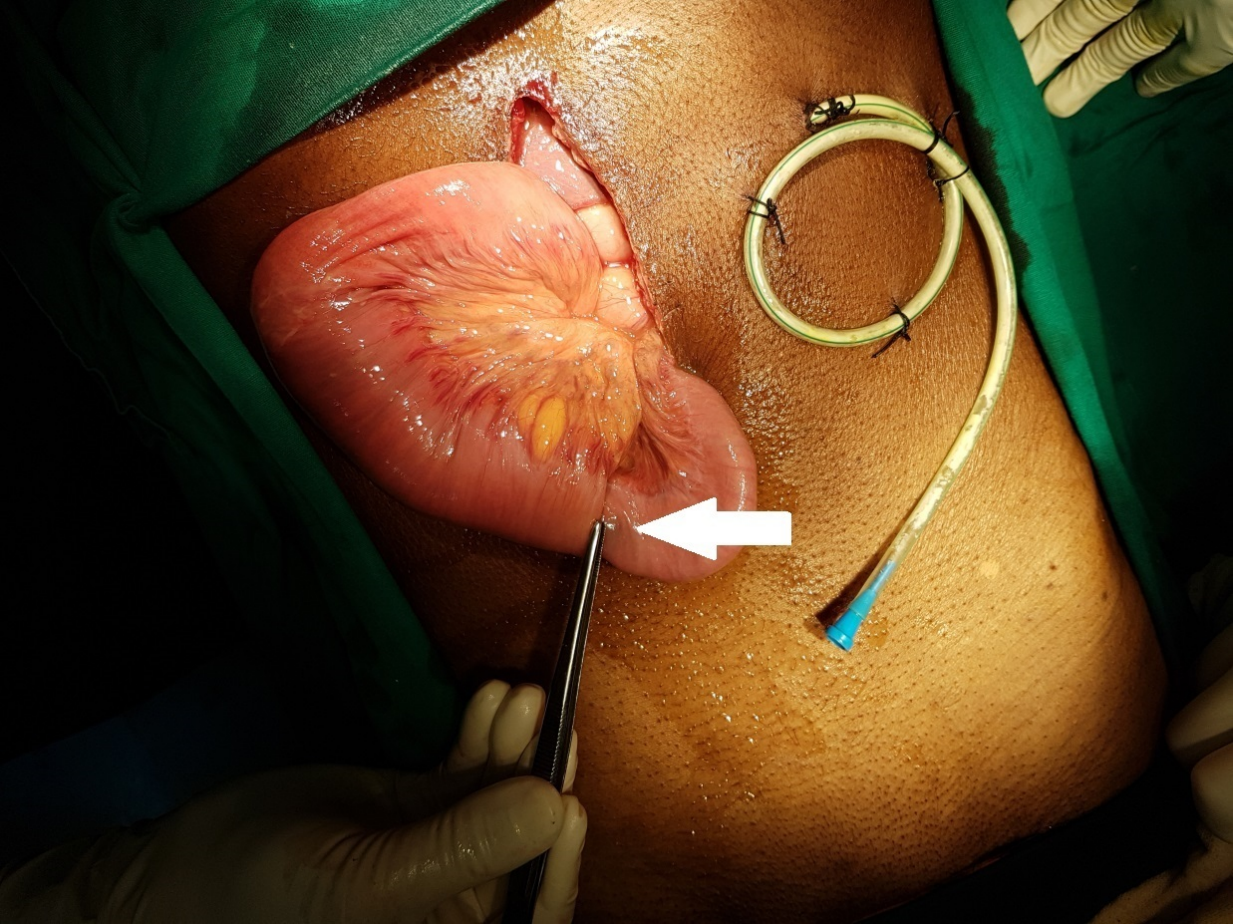
Jejunojejunal intussusception around the FJ tube (white arrow) away from the tip that was reduced FJ - feeding jejunostomy

A 57-year-old male presented with complaints of dysphagia for one month. A UGIE study showed an ulceroproliferative growth from 28 to 35 cm from the incisors. CECT thorax and abdomen demonstrated a growth extending from T6 to T10, abutting the left atrium, and with a greater than 90-degree sectoral contact with the aorta. As the patient was in absolute dysphagia, he underwent feeding jejunostomy by the Witzels technique using a 16 Fr nasogastric tube. Postoperatively, after starting jejunostomy feeds, the patient developed pain abdomen and abdominal distension. An ultrasonography of the abdomen showed a long segment intussusception noted along the feeding jejunostomy tube with proximal dilated jejunal loops and free fluid in the abdomen. On an exploration of the abdominal cavity, there was a 10-cm long segment intussusception 10 cm distal to the FJ fixation to the parietal wall (Figure 4).

The bowel was edematous but healthy and viable. There was a peritubal bile leak at the FJ enterotomy site. The intussusception reduced and the enterotomy site was repaired and refixed to the parietal wall. Postoperatively, the patient had an uneventful recovery.

Case 3

A 24-year-old lady presented with a history suggestive of gastric outlet obstruction. The UGIE study revealed a deformed pylorus suggestive of chronic peptic ulcer disease. The patient underwent truncal vagotomy and loop gastrojejunostomy. Intra-operatively, a 14 Fr enteral feeding tube was passed to the efferent loop for early nasojejunal feeds. Postoperatively, after starting nasojejunal feeds, the patient developed a persistent abdominal pain and bilious vomiting. The patient was explored in view of a suspicion of the efferent loop syndrome. On exploration, there was a short segment intussusception in the efferent limb starting proximal to the tip of the nasojejunal tube. The intussusception was reduced and a side-to-side Brauns jejunojejunostomy was done. Postoperatively, the patient had an uneventful recovery.

Case 4

**Figure 5:**
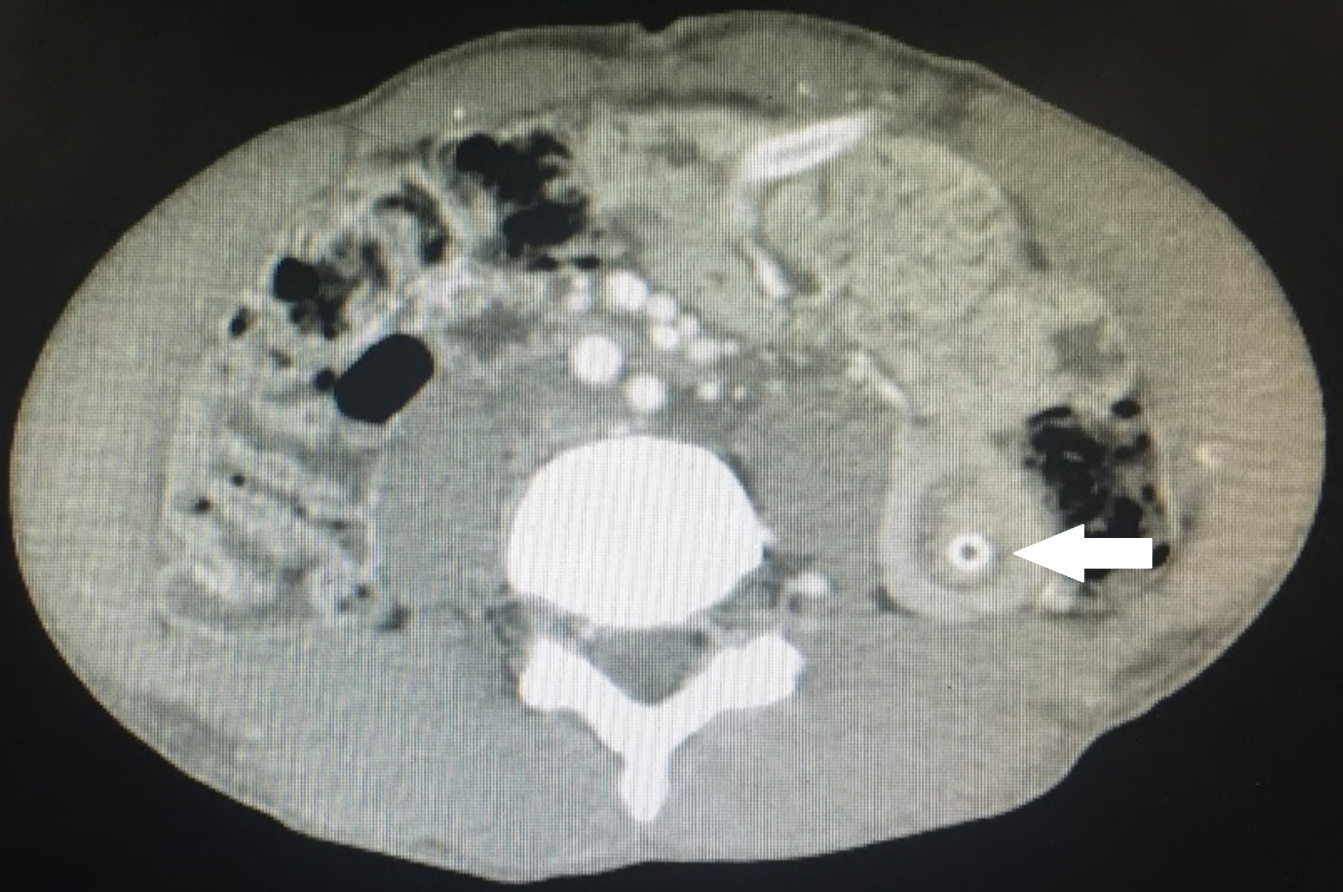
CECT showing the target sign of jejunojejunal intussusception (white arrow), which was conservatively managed CECT: contrast-enhanced computed tomography

A 29-year-old male presented with a history of corrosive ingestion. On the UGIE study, a circumferential esophageal ulceration involving the entire esophagus starting from 17 cm was seen. The patient underwent feeding jejunostomy, as he was not tolerating orals with grade 4 dysphagia. The feeding jejunostomy was done by the Witzels technique using a 14 Fr nasogastric tube. The feeding through FJ was started and the patient was discharged once he tolerated the feeds. After one month, the patient presented with complaints of a colicky type of abdominal pain and multiple episodes of bilious vomiting. The general physical examination revealed that the abdomen was soft. An ultrasonography of the abdomen demonstrated an around 7-cm long jejunojejunal intussusception at the site of insertion of the FJ, which was confirmed with a CECT (Figure 5).

The patient was managed conservatively, as there was no evidence of any peritoneal signs. The feeding jejunostomy was let to a drain and intravenous fluid was given. The symptoms of the patient were improved in three days and FJ feeds were started slowly. The patient tolerated the FJ feeds and then discharged.

## Discussion

Intussusception is defined as the telescoping of the proximal segment of the gastrointestinal tract within the lumen of the adjacent segment. It is a common cause of intestinal obstruction in the pediatric population and is characterized by a classical triad of colicky abdominal pain, red currant jelly stools, and a sausage-shaped abdominal mass. It is a rare entity in adults and accounts for only 5% of the intussusception [2]. The presentation of adult intussusception may be a more chronic or vague abdominal pain (71%), nausea and vomiting (68%), abdominal distension with partial obstruction (45%), or a palpable mass at physical examination [5]. Adult intussusception also differs from the pediatric one in that it is most often associated with a pathologic lead point. The lead point can be benign neoplasms, carcinomas, diverticulum, or strictures [6]. The placement of an intestinal tube for feeding purposes has been rarely known to cause intussusception, accounting for 1% of all cases of intussusception [7]. We described four cases of jejunojejunal intussusception and their management, from which three were caused by the placement of feeding jejunostomy tubes by the Witzels technique and one caused by the placement of the feeding tube in the efferent limb after gastrojejunostomy.

Different theories have been proposed for intussusception caused by the placement of feeding tubes [8-9]:

1. The tip of the feeding tube can act as the leading point and drag the proximal segment over the distal segment during a peristaltic wave.

2. Retrograde peristalsis of the jejunum during episodes of vomiting.

3. The injecting force from the pump during feeding can also drag the bowel, causing intussusception.

4. Poorly built patients have reduced fatty tissue in the omentum and mesentery, which may allow the free movement of the bowel causing a predisposition to intussusception.

5. An increased caliber or a longer length of the feeding tube used in the bowel segment may produce intussusception due to distal tip migration [4].

The exact mechanism of intussusception in our patients is not clearly understood, as the lead point was not at the tip of the feeding tube on exploration. The lead point was proximal to the tip of the feeding tube placed. So, the assumption is the tip would have acted as the initial lead point and, later, migrated proximally due to peristalsis.

To diagnose a case of intussusception, the initial imaging modality is an abdominal ultrasound. But the investigation of choice for identifying an intussusception in adults is the CECT abdomen. The features of intussusception in a CECT are the presence of intraluminal bowel segments with or without the presence of fat and mesenteric vessels and a clear doughnut-shaped mass due to edema, in the transverse views (target sign) [10].

Intussusception caused by feeding tubes usually resolves spontaneously with operative intervention needed in a few cases. It can be managed by changing the tube to a standard or short tube without a distal pigtail, reduction by injecting air or contrast through the tube, or by the exchange of the tube over a guidewire under fluoroscopic guidance [8]. But, these measures are helpful only in cases that show transient radiological evidence of intussusception. Operative intervention was required in three of our patients, as the symptoms did not resolve when they were managed conservatively. Wu et al. reported a jejunojejunal intussusception following feeding jejunostomy by the Witzels technique managed by operative reduction and recommends operative treatment for all cases presenting with signs of obstruction [9]. The removal of the jejunostomy tube was not required after reduction and all our patients had an uneventful postoperative period without any recurrence.

## Conclusions

Jejunojejunal intussusception is a very rare complication after the placement of feeding jejunostomy tubes and needs a high index of suspicion. So, this rare possibility should be kept in mind in any postoperative patients with FJ presenting with intestinal obstruction. CECT is highly sensitive for detecting intussusception. Operative intervention is required in all patients who don’t resolve with expectant management. The removal of the feeding jejunostomy tube is not necessary to prevent a recurrence.
